# Aspirin Exacerbated Respiratory Disease and Nasal Polyp Phenotyping

**DOI:** 10.22037/ijpr.2021.114924.15113

**Published:** 2021

**Authors:** Reza Kaboodkhani, Amirreza Bolkheir, Hossein Esmaeilzadeh, Mohammad Faramarzi, Mohammadjavad Ashraf, Milad Hosseinialhashemi, Negar Mortazavi, Narjes Ebrahimi

**Affiliations:** a *Otolaryngology Research Center, Shiraz University of Medical Sciences, Shiraz, Iran. *; b *Allergy Research Center, Shiraz University of Medical Sciences, Shiraz, Iran. *; c *Department of Allergy and Clinical Immunology, Medical School, Shiraz University of Medical Sciences, Shiraz, Iran. *; d *Department of Pathology, School of Medicine, Shiraz University of Medical Sciences, Shiraz, Iran. *; e *Student Research Committee, Shiraz University of Medical Sciences, Shiraz, Iran. *; f *Department of Clinical Pharmacy, School of Pharmacy, Shiraz University of Medical Sciences, Shiraz, Iran.*; 1 *1R. K. and A. B. contributed equally to this work.*

**Keywords:** AERD, Eosinophilic polyp, Hypersensitivity, Neutrophilic polyp, Sinusitis

## Abstract

Aspirin exacerbated respiratory disease (AERD) is known by the triad of chronic rhinosinusitis with nasal polyposis (CRSwNP), aspirin hypersensitivity, and asthma, but its etiology and physiopathogenesis are still unclear. This cross-sectional study was designed to investigate allergy and inflammatory cells (neutrophils *vs.* eosinophils) dominancy in nasal polyp tissue of patients with AERD compared to non-AERD patients. CRSwNP patients scheduled for endoscopic sinus surgery were recruited in this study. Nasal polyp tissue was analyzed for infiltrating cells, and Eosinophil dominant and neutrophil dominant polyps were determined. AERD was confirmed by oral aspirin challenge (OAC). Demographics data; history of asthma, exacerbation by using NSAIDs, routine use of aspirin, type of surgery (primary or revision), and results of skin prick test and spirometry were recorded. Pathology results and contributing factors compared between AERD and non-AERD patients. Sixty-five patients (39 women, 26 men) were enrolled in the study (mean age 38.83 ± 12.43 years). Thirty (46%) patients had positive OAC tests. Totally 41 patients (63.1%) had eosinophilic polyps. 80% of patients with eosinophilic polyp had positive OAC and were AERD (*P *< 0.05). There was no significant difference in demographics, revision surgery, and concomitant asthma between AERD and non-AERD groups (*P *> 0.05). The positive skin prick test was higher in AERD and also in eosinophilic polyp patients, but it was not statistically significant (*P* = 0.086 and *P *= 0.177). Eosinophilic polyps are more common in AERD. A positive skin prick test is associated with AERD and eosinophilic polyp.

## Introduction

Chronic rhinosinusitis (CRS), affecting a large population worldwide (about 10%) (1). Diagnosis of CRS includes subjective symptoms, such as nasal congestion, rhinorrhea, hyposmia, and facial pain persisting more than 12 weeks; and objective symptoms are confirmed by paranasal sinus computerized tomography (CT scan). CRS affects patients’ health-related aspects, such as sleep and mood, and is associated with high indirect costs (2). About one–third of cases with CRS are associated with nasal polyps, called chronic rhinosinusitis with nasal polyp (CRSwNP), with a peak incidence in middle age (3) and mainly affecting men; however, women are supposed to have more severe disease (4).

Management of CRSwNP includes medical treatment in mild cases and surgery in severe cases. There are several factors including the presence of concomitant diseases, which result in poor treatment outcomes (5). About 30- 40% of CRSwNP patients may have aspirin-exacerbated respiratory disease (AERD) characterized by hypersensitivity to aspirin and non-steroid anti-inflammatory drugs (NSAIDs), asthma and nasal polyposis (6). Patients with concomitant CRSwNP and AERD have more severe disease, higher recurrence of nasal polyps, and frequent need for sinus surgery (4, 7) Therefore, it is valuable to study factors associated with AERD in patients with CRSwNP like an allergy. A mutual correlation is also observed between CRSwNP and asthma. Patients with CRSwNP are prone to severe asthma and asthmatic patients have a higher incidence of CRSwNP, especially in AERD (8). Coexistence of CRSwNP and asthma, entitled “United Airway Disease”, resulted in poor treatment outcomes and guided research towards studying the underlying pathophysiology of these conditions to help better understanding these diseases, their association, and more appropriate treatment options (9).

Studies focusing on the pathogenesis of CRSwNP and AERD have suggested several mechanisms as the underlying causes of CRSwNP, while it is generally accepted that chronic inflammation induced by dysregulated immune response is the main pathogenesis of CRSwNP (10). As the main role of sinonasal mucosal tissue is functioning as the epithelial immunological barrier, the physical or functional defect of this barrier results in dysregulation of both innate and adaptive immune responses. These defects result in the release of several inflammatory markers, such as interferon (IFN–γ), tumor necrosis factor (TNF–α), transforming growth factor (TGF–β), and interleukins (ILs) (11, 12). Infiltration of eosinophils and neutrophils with inflammatory and tissue remodeling effects is another important finding in patients with CRSwNP, associated with higher disease severity and a greater chance of recurrence (13). However, different rates of eosinophilic polyps have been reported in different countries for example more neutrophilic nasal polyp in Asia and more eosinophilic polyp in Europe and North America (14). Accordingly, it has been suggested that infiltration of eosinophils and neutrophils may vary based on the patients’ race/ethnicity.

In a study conducted in Iran, 48.8% of the patients with CRSwNP were diagnosed with AERD. The association of AERD with nasal polyp surgery, asthma and history of ASA hypersensitivity was reported (4). However, the eosinophil or neutrophil dominancy of polyp tissue has not been studied in the Iranian population yet. In addition, the association of eosinophil or neutrophil dominancy with AERD and asthma and allergy in patients with CRSwNP has also not been established in Iran. Therefore, in this study, we aimed to examine the infiltration of eosinophils and neutrophils in nasal polyp tissues and allergy sensitizations. AERD that confirmed by the oral aspirin challenge test.

## Experimental

*Study design*

This study was designed as a cross-sectional study, performed at Otorhinolaryngology Department and Allergy Research Center. Patients who were referred from March 2017-2020 for endoscopic sinus surgery enrolled in the study and were referred to the Allergy Clinic for an oral aspirin challenge (OAC) test, two months after surgery. A skin prick test was performed to investigate allergic sensitization to the most prevalent aeroallergens in Iran. The Ethics Committee of Shiraz University of Medical Sciences approved the protocol of the study (number: 17462). Written informed consent was obtained from all participants.

Patients with a diagnosis of CRSwNP that had poor response to medication therapy in 6 weeks and were scheduled for functional endoscopic sinus surgery (FESS) were recruited in this study. CRSwNP diagnosis was based on the European position paper on rhinosinusitis and nasal polyps (1).

Exclusion criteria: age ≤ 18 years, pregnancy, hematologic abnormality and bleeding tendency, unstable asthma or cardiopulmonary condition, history of gastrointestinal bleeding and history of NSAID anaphylaxis. Patients who were not willing to do the aspirin challenge test were excluded from the study.

*Data collection*

Patients’ demographics, such as age and sex; history of asthma, exacerbation of symptoms by using NSAIDs, history of routine use of aspirin (ASA), history of previous endoscopic sinus surgery, and results of allergy skin prick test were recorded.

All the participants underwent a standard oral aspirin challenge (OAC) test described by Nabavi *et al.* (4) two months after FESS polypectomy. OAC was performed in the outpatient Allergy Clinic by increasing doses of aspirin (20, 40, 80, 160, 320 mg) every 90 minutes. Before the test, baseline FEV_1_ was measured, and if the baseline FEV_1_ was > 70% of the expected value, the patient was considered stable and prepared for the test. OAC test was positive and stopped if one of the following conditions occurred: 20% decrease in FEV1, any clinical symptoms during the test such as extra bronchial symptoms (severe congestion, runny nose, urticaria, and cough) and a 15% decrease in FEV1. Patients with positive OAC were considered as AERD.

All patients underwent endoscopic sinus surgery by an expert otorhinolaryngologist. A biopsy sample was taken from the polyp tissue and sent for pathological examination. In the laboratory, first, the samples were kept in paraformaldehyde at 4 ºC overnight, and they were transferred to buffered phosphate saline the day after, and finally in 70% ethanol until suspension in paraffin. Afterward, the tissues were sectioned, stained with Hematoxylin and Eosin (H&E), and examined by the pathologist using a light microscope. With a magnification of ×400, the number of eosinophils and neutrophils were counted in 10 sections of the tissue and their mean value was recorded. The dominancy of eosinophils or neutrophils was recognized by the pathologist and the polyp was considered as eosinophilic and neutrophilic.

*Statistical analysis*

All the collected data were analyzed by the statistical software IBM SPSS Statistics for Windows version 21.0 (IBM Corp. 2012. Armonk, NY: IBM Corp.). Descriptive results of nominal variables were presented by frequency (percentage) and that of numeric variables by mean ± standard deviation (SD). One–sample Kolmogorov–Smirnov test was used to assess the normal distribution of data and equality of variances tested by Levene’s test. For comparison of continuous variables, *t*-test or Mann–Whitney U test was applied, whenever the data did not appear to have normal distribution or when the assumption of equal variances violated across the study groups. Categorical variables, on the other hand, were compared using Chi-square test. The difference between groups was considered statistically significant for *P* < 0.05.

## Results

Sixty-five patients with the mean age of 38.83 ± 12.43 years were enrolled in this study. The minimum age was 19 and the maximum was 68 years old. Of all patients, 39 (60%) patients were female and 26 (40%) were male.

The results of the OAC test showed that 30 patients (46%) were AERD (sensitive to aspirin) and 35 (54%) were non-AERD. In the AERD group, the mean dose resulting in positive OAC was 143.166 ± 119.36 mg. The mean age and sex distribution of patients were not different between the groups with and without aspirin sensitivity (*P* > 0.05) (Table 1).

The type of polyp was eosinophilic in 41 patients (63.1%) and neutrophilic in the remaining. There was a significant difference between the groups of AERD and non-AERD in terms of polyp type. As shown in table 1, 80% of AERD patients had eosinophilic polyp (*P* = 0.011).

The results of skin tests were recorded and positive results were observed in 42 patients (65%). The percent of positive tests was higher in the AERD patients group compared to non-AERD (73% *vs*. 57%), but without statistically significant difference (*P* = 0.086) (Table 1). 29 out of 41 patients (70%) with eosinophilic polyps had positive skin prick test results, however, the difference was not statistically significant compared to patients with neutrophilic polyp (54%, 13 out of 24 patients) (*P* = 0.177). Of all patients, 23 patients (35.4%) had concomitant asthma, however, its frequency was not different between the groups with and without aspirin sensitivity (*P* = 0.118) (Table 1).

Of all patients, only nine (13.8%) had a positive history of exacerbations after using NSAIDs and only six patients (9.2%) had a positive history of routine aspirin consumption, without significant difference between AERD and non-AERD groups in these regards (*P* > 0.05) (Table 1). Moreover, 14 (21.5%) underwent revision surgery and the frequency of revision surgery was not different between the groups (*P* = 0.771) (Table 1).

## Discussion

In this study, we analyzed inflammatory cells in nasal polyp tissue of 65 CRSwNP patients after FESS surgery and determined aspirin hypersensitivity and contributing factors. OAC was performed 2 months after surgery to confirm AERD. Eighty percent of AERD patients had an eosinophilic polyp and a positive skin prick test was higher in patients with an eosinophilic polyp.

The results of the OAC test showed that 46.15% of CRSwNP patients had AERD with a mean positive OAC dose of 143.166 mg, while only 13.8% reported a self-positive history of symptoms exacerbation after using NSAIDs. The discrepancy in the frequency of subjective (self–report) and objective (OAC test) frequency of AERD in the present study that is compatible with previous research by authors (4) could be due to the frequency of routine NSAID consumption by patients and the point that main diagnostic test for AERD is OAC.

The association of AERD with CRSwNP has been well established (15); however, there are few studies addressing the frequency of AERD in CRSwNP by OAC. Nabavi *et al*. showed AERD in 48.8% of CRSwNP patients at a mean aspirin dose of 121.5 mg (4). The frequency of AERD reported in their study is similar to that of ours, while the mean dose of aspirin in their study was lower than in this study. The different frequency of AERD reported in literature could be due to the different genetics and different diagnostic method as well as different severity of disease in the study populations. In the present study, we included patients with indications for endoscopic sinus surgery, who were supposed to have a more severe disease condition than overall patients with CRSwNP, studied in previous studies (16, 17).

Inflammatory pathogenesis is suspected as one of the main predisposing factors in different clinical presentations and responses to treatment between AERD and non-AERD patients (18). Accordingly, we examined the polyp tissue taken during surgery in patients with CRSwNP and compared the cellular dominancy in the tissue samples. The results of the pathological examination showed nasal polyp with eosinophil dominancy in 63.1% of patients, which indicated atopy and allergy as one of the main nasal polyp pathogenesis. Based on the results, the frequency of eosinophil or neutrophil dominancy was different between the patients with and without AERD. Most patients with AERD (80%) had eosinophil dominancy, while about half of non-AERD patients had eosinophil dominancy (48.6%) with a significant difference between the groups in this regard. These findings are consistent with the results of previous studies indicating eosinophilic type as the most common type of CRSwNP in AERD (19). Recent researches have demonstrated different inflammatory signatures of CRSwNP; with less eosinophilic and more neutrophilic inflammation found in Asia compared with Europe and North America (20). It is the first time in our country that we work on inflammatory cells of nasal polyp and although our study is conducted in Asia, it seems that our findings are more compatible with non-Asian findings.

The results of this study showed neutrophilic dominancy in 20% of aspirin-sensitive patients and about half in non–aspirin-sensitive patients. Besides the role of neutrophils in the disease (20), eosinophil cells also exist in patients with neutrophil dominancy in polyp tissue, which may have specific roles in the disease. In a Japanese study, the researchers classified patients with CRSwNP into eosinophilic (N = 42), neutrophilic (N = 27), and non–eosinophilic non–neutrophilic (N = 61) types and reported higher IgE values and expressions of Eotaxin, IL–17A, and CD68 in eosinophilic type, which suggest different pathophysiology between these groups (21). The association of tissue eosinophilia with CRSwNP severity is still controversial as some researchers have proposed tissue eosinophilia as a predictor of poor treatment outcome and higher recurrence rate (22), while others have rejected such association (23), and some studies have suggested poorer treatment outcome in the neutrophilic type (24). While results are mainly observer-based and there are no standard criteria for determining cell dominancy, further research is required in this regard (25).

In the present study, 35.4% of patients with CRSwNP had concomitant asthma. The frequency of asthma in our patients was higher than a previous Iranian study that reported asthma in 24% of patients with CRSwNP (26). This difference could be first due to the different disease severity and type (allergic), as more severe CRSwNP (patients with CRSwNP indicated for endoscopic sinus surgery were included) and allergic-type CRSwNP in our study could be associated with a higher prevalence of asthma (27). Another important reason for the different frequency of asthma in patients with CRSwNP is that this value depends on the prevalence of asthma in the general population. Fan *et al*. (28) have reported the prevalence of asthma in 2–3% of patients with CRSwNP, which is supposed to be due to the low prevalence of asthma and atopy in this country. In addition, we studied the association of asthma with AERD in patients with CRSwNP. As the results indicated, although the prevalence of asthma was higher in the AERD group in our study, there was no statistically significant difference in the frequency of asthma between patients with and without AERD, which is contrary to the results of previous studies suggesting an association of asthma with AERD, which can be due to different sample size (15, 29). Moreover, the results of our study showed higher positive skin prick test in both AERD and eosinophilic polyp patients, but there was no statistically significant difference in the skin test results between AERD and non-AERD groups, and eosinophilic and neutrophilic groups, as well. Although the role of allergy in AERD has not been established yet, it seems that atopy is associated with AERD (30). AERD and allergic polyp are associated with recurrent and refractory nasal polyp (13) that is not compatible with our finding that showed no association between AERD and recurrence of the polyp. Also, in our study, there was not any association between eosinophilic polyp and recurrence of nasal polyp. Aspirin desensitization can help with reducing the recurrence of nasal polyp in AERD by changing the nasal polyp tissue (31-33). Yet, further research is required to confirm this issue. These discrepancies can be due to low sample size and genetic differences. Differences in genetics and the effect of epigenetics emphasize the difference in results of studies and the need for studies in different populations (4, 34).

One of the main strengths of the present study was the objective assessment of asthma and aspirin sensitivity in patients with CRSwNP, and pathological examination of nasal polyp tissue for the first time in the country. However, this study could have some limitations. The sample size of the study was small, although we extended the duration of sampling. Another limitation was that we did not follow patients for the long term after surgery to observe the effect of surgical treatment on the disease conditions, which can be a topic for further studies.

Further studies are required to indicate the clinical significance of eosinophilic dominant polyps and aspirin sensitivity in patients with CRSwNP and their impact on the treatment outcome of patients.

In conclusion, the results of the present study confirmed that nasal polyp with eosinophilic dominancy is more prevalent in AERD patients. However, there was no association between asthma, recurrence of polyp, and AERD. A positive skin prick test is associated with AERD and eosinophilic polyp. Our findings can help in phenotyping the patients with CRSwNP and justify the different clinical responses of patients with CRSwNP.

**Table 1:** Demographic and clinical characteristics of patients with and without aspirin sensitivity

**Variable**	**Category**	**Patients with aspirin sensitivity (N = 30)**	**Patients without aspirin sensitivity (N = 35)**	***P*** **-value**
Age (years), mean ± SD		41.27 ± 11.74	36.74 ± 12.78	0.145^*^
Sex, No. (frequency)	Female	21 (70%)	18 (51.4%)	0.204^†^
	Male	9 (30%)	17 (48.6%)	
Polyp type, No. (frequency)	Eosinophilic	24 (80%)	17 (48.6%)	0.011^†^
	Neutrophilic	6 (20%)	18 (51.4%)	
Skin test results, No. (frequency)	Positive	22 (73%)	20 (57%)	0.086
	Negative	8 (27%)	15 (43%)	
Concomitant asthma, No. (frequency)	Yes	14 (46.7%)	9 (25.7%)	0.118^†^
	No	16 (53.3%)	26 (74.3%)	
History of exacerbation after using NSAIDs^‡^, No. (frequency)	Yes	7 (23.3%)	2 (5.7%)	0.069^†^
	No	23 (76.7%)	33 (94.3%)	
History of routine aspirin consumption, No. (frequency)	Yes	5 (16.7%)	1 (2.9%)	0.087
	No	25 (83.3%)	34 (97.1%)	
Revision surgery, No. (frequency)	Yes	7 (23.3%)	7 (20%)	0.771
	No	23 (76.7%)	28(80%)	

^*^The results of *t*-test,^ †^The results of Chi-square test,^ ‡^NSAIDs; Non-steroid anti-inflammatory drugs.

## Conflict of interest

 The authors declare that they have no conflict of interest.

## Authors’ Contributions

R. kaboodkhani and M. Faramarzi as professors and A. Bolkheir as a resident of ENT contributed to the study design, diagnosis of nasal polyp and surgery. M. Ashraf as a pathologist contributed to polyp tissue sample analysis, study design, data analysis and manuscript drafting. H. Esmaeilzadeh as an allergist and N. Mortazavi as clinical pharmacist performed oral aspirin challenge and participated in study design, data registry, data analysis and manuscript drafting. M. Hosseinialhashemi as a medical intern transferred samples, helped with laboratory analysis, data registering and analysis, and writing manuscripts. N. Ebrahimi contributed to study design, data analysis, manuscript drafting and revising. This study is extracted from A. Bolkheir’s thesis on ENT residency.
